# Farnesyl Diphosphate Synthase Promotes Proliferation of Hepatocellular Carcinoma Cells by Interacting With Glucose‐6‐Phosphate Dehydrogenase

**DOI:** 10.1002/cam4.71620

**Published:** 2026-02-10

**Authors:** Jingfeng Liu, Yisheng Zhu, Jiyang Lv, Xiaohao Hu, Yan Zhong

**Affiliations:** ^1^ Shenzhen Key Laboratory of Immunity and Inflammatory Diseases, Department of Rheumatism and Immunology Peking University Shenzhen Hospital Shenzhen China; ^2^ Institute of Biomedicine and Biotechnology, Shenzhen Institute of Advanced Technology Chinese Academy of Science Shenzhen China; ^3^ Department of Pathology, Shenzhen Hospital Southern Medical University Shenzhen China

**Keywords:** cell apoptosis, cell proliferation, cholesterol metabolism, glycolysis, hepatocellular carcinoma

## Abstract

**Background:**

Hepatocellular carcinoma (HCC) is a highly aggressive malignancy characterized by metabolic reprogramming that supports tumour growth and survival. This study identifies farnesyl diphosphate synthase (FDPs), a key enzyme in the mevalonate pathway, as a critical regulator of HCC proliferation and apoptosis.

**Methods:**

We applied bioinformatics analysis through TCGA and GSE database to identify the expression of FDPs within HCC patients. Then, mechanistic studies were conducted including Western blots, apoptosis assay, RT‐qPCR, rescue assay, RNA‐sequencing, in vivo study to prove the role of FDPs in regulating HCC progression.

**Results:**

FDPs was found to be significantly upregulated in HCC tissues, and its down‐regulation promotes tumour cell apoptosis while inhibiting tumour cell proliferation in vitro and in vivo. Mechanistically, we identified FDPs regulate glucose‐6‐phosphate dehydrogenase (G6PD) by RNA sequencing, bioinformatics prediction, and rescue experiments, indicating its involvement in glycolysis regulation in tumour cells. The identification of this FDPs–G6PD axis suggests a novel metabolic pathway contributing to HCC development.

**Conclusion:**

In summary, this study highlights FDPs play an essential oncogenic role in HCC, linking it to metabolic reprogramming and tumour survival. These findings establish FDPs as a promising therapeutic target, offering a foundation for further exploration of its regulatory mechanisms and potential clinical applications.

**Abbreviations**
ECAR: Extracellular acidification rate
FDPs: Farnesyl diphosphate synthase
G6PD: Glucose‐6‐phosphate dehydrogenase
GO: Gene Ontology
HCC: Hepatocellular carcinoma
ICIs: Immune checkpoint inhibitors
KEGG: Kyoto Encyclopedia of Genes and Genomes
LDLR: Low‐density lipoprotein receptor
LXR: Liver X receptor
NAFLD: Non‐alcoholic fatty liver disease
OCR: Oxygen consumption rate
PPP: Pentose phosphate pathway
SQLE: Squalene epoxidase
SREBP2: Sterol regulatory element‐binding protein 2

## 1 Introduction

Hepatocellular carcinoma (HCC) is the most prevalent primary liver malignancy [1, 2], ranking as a leading cause of cancer‐related morbidity and mortality worldwide [1, 2]. In recent years, advancements in therapeutic strategies such as systemic chemotherapy and immune checkpoint inhibitors (ICIs) have gained a big amount of attention [3, 4]. ICIs targeting pathways like PD‐1, PD‐L1 and CTLA‐4 have shown potential in HCC through improved immune‐mediated tumor suppression [5, 6, 7]. Despite these advances, clinical challenges such as primary and acquired drug resistance, limited response rates, and high recurrence rates persist, hampering the effectiveness of current treatments [4, 8]. Furthermore, resistance linked to genetic and metabolic reprogramming in HCC has significantly limited the ability to sustain durable therapeutic responses [9, 10]. Therefore, there is an urgent need to identify novel molecular targets and elucidate key cellular mechanisms driving HCC progression to develop effective anti‐cancer treatments [11].

Cholesterol is a vital component of the plasma membrane, supporting cell proliferation, growth, and structural integrity [12, 13]. Recent studies have highlighted the significance of reprogramming cholesterol metabolism in cancer progression, revealing that accelerated synthesis, abnormal uptake, and impaired efflux are closely associated with tumorigenesis in various types of cancer [14, 15]. For example, squalene epoxidase (SQLE) has been shown to drive prostate cancer progression by enhancing cholesterol biosynthesis [16]. Similarly, in glioblastoma, cholesterol uptake through low‐density lipoprotein receptor (LDLR) supports the growth of tumor stem cells, while abnormal cholesterol accumulation contributes to epithelial–mesenchymal transition in pancreatic cancer [17]. Moreover, dietary cholesterol has been implicated in promoting colorectal cancer progression, showcasing the impact of exogenous cholesterol on tumor biology [18]. In addition to supporting tumor cell growth, cholesterol and its derivatives like oxysterols influence the immune microenvironment of cancer. Oxysterols modulate transcription factors such as sterol regulatory element‐binding protein 2 (SREBP2) and liver X receptor (LXR) to alter T‐cell functionality, contributing to immune evasion in melanoma [19]. In addition, several hormones, such as thyroid hormone, are reported to regulate cholesterol metabolism in the liver [20, 21]. Hypothyroidism increased the level of cholesterol and causes non‐alcoholic fatty liver disease (NAFLD), which is strongly related to liver malignancy [20]. Therefore, given the critical role of the liver in cholesterol biosynthesis and regulation, a detailed understanding of the contribution of cholesterol metabolism in HCC progression is urgently needed.

Farnesyl diphosphate synthase (FDPs), an integral enzyme in the mevalonate pathway involved in cholesterol biosynthesis, has emerged as a crucial regulator of metabolic homeostasis in cancer [22]. For instance, FDPs regulate GTPase/AKT axis activity in PTEN‐deficient prostate cancer in promoting cancer cell proliferation [23]. Moreover, FDPs have been implicated in the regulation of Rho family proteins, such as RhoB. FDPs modulate RhoB protein stability and integrin β1 localization to promote bladder cancer metastasis [24]. In the tumor microenvironment, dysregulated FDPs activity can drive cellular processes critical for cancer cell survival and proliferation, including enhanced membrane fluidity, energy production, and signal transduction [25, 26]. However, the specific role of FDPs in promoting hepatocellular carcinoma remains unclear. Targeting FDPs could disrupt multiple downstream pathways simultaneously, offering a novel alternative for the development of anti‐cancer strategies in HCC.

In this study, we demonstrated the role of FDPs in regulating tumorigenesis of HCC. By means of bioinformatics, RNA‐seq analysis, in vitro biological and in vivo xenografts model detection, we obtained mechanistic insights into how FPDs regulate HCC cancer cell proliferation and apoptosis by regulating G6PD protein, which might relate to tumor glycolysis. These data may afford the opportunity to develop new therapeutic approaches for HCC.

## 2 Materials and Methods

### 2.1 Cell Culture

Human liver cancer cell line SK‐HEP1 and HepG2 were purchased from ATCC. Cells were cultured in Dulbecco's Modified Eagle's Medium (DMEM) (Gibco, USA) containing 10% fetal bovine serum (HyColne, Utah, USA). Cells were cultured at 37°C in a humidified atmosphere containing 5% CO2. Mycoplasma contamination was excluded.

### 2.2 Cell Proliferation Assay

As for CCK‐8 assay, indicated cells were cultured in 96‐well culture plates (5.0 × 10^3^ cells/well) overnight. Then CCK‐8 reagent was added to each well at indicated time point (24, 48, 72 and 96 h after seeding) followed by incubation for 1 h. Absorbance was measured by EnSpire Multimode Plate Reader. The absorbance of control cells was taken as 100%. As for cell cycle detection, indicated cells were incubated 48 h before harvesting and stained by cell cycle staining kit (BD, 558662). The cell cycle ratio of indicated cells was tested by flow cytometry. As for cell colony formation assay, indicated cells were cultured in 6‐well culture plates (200 cells/well) 14 days before fixing and staining with 5% Giemsa solution (Sigma, 32,884). Cell colonies were photographed and counted.

### 2.3 Cell Apoptosis Assay

Indicated cells were harvested and stained using the AnnexinV‐FITC Apoptosis Kit (BD, 556547). Then the collected cells were analyzed by a FACS machine (BD LSRFOTESSA). The FACS Data were further processed using Flow‐Jo software.

### 2.4 Stable FDPs Knock‐Down Establishment

To establish a stable FDPs knock‐down cell line, shRNA targeting FDPs was used. Oligos targeting FDPs showed as follows: shFDPs #1: CACCGCCATTGGAGGCAAGTATAACCGAAGTTATACTTGCCTCCAATGGC or shFDPs #2 CACCGGTTTGACGGTGGTAGTAGCACGAATGCTACTACCACCGTCAAACC. Oligomers were designed, annealed, and inserted into PLKO.1 puro according to Addgene's pLKO.1 protocol. Lentivirus construction and infection was performed as mentioned before [27]. Briefly, 1 day before the viral infection, cells were plated into a six‐well plate at a density of 1 × 10^5^ cells/well and transduced in the presence of 5 μg/mL polybrene for 72 h. The cells were selected for puromycin resistance (2 μg/mL) for 7 days. Protein expression was further determined by western blots.

### 2.5 G6PD Overexpression

The coding sequence (CDS) of G6PD (NM_000402.4) was cloned by PCR and gibson assembly into p‐EF1α vector (Addgene) following the instructions [28]. G6PD overexpressing cells were selected for BSD resistance (2 μg/mL) for 7 days.

### 2.6 Western Blots Analysis

Indicated whole‐cell extracts were prepared and separated by 8%–12% SDS‐PAGE and transferred to nitrocellulose membranes (Millipore). For the immunoprecipitation assay, the cells were lysed with IP lysis buffer (Thermo Fisher Scientific) containing protease inhibitor cocktail (Thermo Fisher Scientific) on ice for 30 min. After centrifugation at 12,000 × g for 20 min at 4°C, the supernatants were immunoprecipitated with antibodies followed by incubating with magnetic protein A/G beads (Pierce) for 4 h. Antibodies including FDPs (ab153805, Abcam), G6PD (#DF6444, Affinity), P21 (AF6290, Affinity), H2AX (#7631, CST), γ‐H2AX (#9718, CST), and β‐Actin (4970 T, CST) were used in this study.

### 2.7 RT‐qPCR

Total RNA of indicated cells was isolated and reversely transcribed to cDNA. Then RT‐qPCR was performed as we previously described [27]. In this study, the primer sequences used in RT‐qPCR are shown in Table 1.

**TABLE 1:** The primers used in this study.

Genes	Primers (5′‐3′)
PUMA	F: ACGACCTCAACGCACAGTACGA R: CCTAATTGGGCTCCATCTCGGG
P21	F: ACCTGGAGACTCTCAGGGTCG R: TTAGGGCTTCCTCTTGGAGAAGAT
PERP	F: TCATCCTGTGCATCTGCTTC
	R: GGGTTATCGTGAAGCCTGAA
NOXA	F: ACCAAGCCGGATTTGCGATT R: ACTTGCACTTGTTCCTCGTGG
CDK1	F: TGAGGTAGTAACACTCTGGTA R: ATGCTAGGCTTCCTGGTT
Cyclin B1	F: TTGGTTGATACTGCCTCTC R: TCTGACTGCTTGCTCTTC
FDPs	F: GCTTTCTACTCCTTCTACCTTCC R: CCCCAAAGAGGTCAAGGTAATC
G6PD	F: AGAACATTCACGAGTCCTGC R: GTGGTCGATGCGGTAGATC
β‐Actin (human)	F: TCGTGCGTGACATTAAGG R: AAGGAAGGCTGGAAGAGT

### 2.8 Tumor Xenograft In Vivo Analysis

For tumor formation of indicated HepG2 in NSG mice, 1.0 × 10^6^ control HepG2 cells and inducible FPDs‐KD HepG2 cells were harvested, washed twice with PBS, suspended in PBS with 50% Matrigel, and subcutaneously injected into hind legs of NSG mice. DOX was applied at Day10 after transplantation. The tumors were collected and weighed 26 days later. All animal experiments were approved by the Institutional Animal Care and Use Committee of Southern Medical University.

### 2.9 RNA Sequencing and Analysis

RNA sequencing and data analysis were performed as mentioned before [27]. Briefly, RNA samples were generated and purity checked using kaiaoK5500Spectrophotometer (Kaiao, Beijing, China), followed by sequencing libraries establishment using NEBNext Ultra RNA Library Prep Kit for Illumina (#E7530L, NEB, USA). Paired‐end sequencing was completed on an Illumina HiSeq system. GRCh38 genomes as the reference and the annotation files were downloaded from ENSEMBL database (http://www.ensembl.org/index.html). Bowtie2 v2.2.3 (https://bowtie‐bio.sourceforge.net/bowtie2/index.shtml) was used for building the genome index, and clean data was then aligned to the reference genome using HISAT2 v2.1.0 (https://daehwankimlab.github.io/hisat2/). Reads count for each gene in each sample was counted by Featurecounts [29]. The differentially expressed genes were analyzed by DESeq2. The adjusted *p* < 0.05 was set as the cut‐off value.

### 2.10 Statics Analysis

Statistics were performed as described in our previous methods [27]. Briefly, SPSS version 20 was utilized to perform statistical analyses, and Prism 9 was utilized for image generation. An unpaired two‐tailed *t*‐test was conducted for comparisons between two groups with equal sample sizes, whereas a paired two‐tailed *t*‐test was utilized for analyzing two groups of paired samples. All experiments were biologically replicated a minimum of three times. Data are presented as the mean ± SD. All values presented are the mean ± SD. *p* < 0.05 and *p* < 0.01 are considered statistically significant.

## 3 Results

### 3.1 High Expression of FDPs is Related to HCC Progression

**FIGURE 1:**
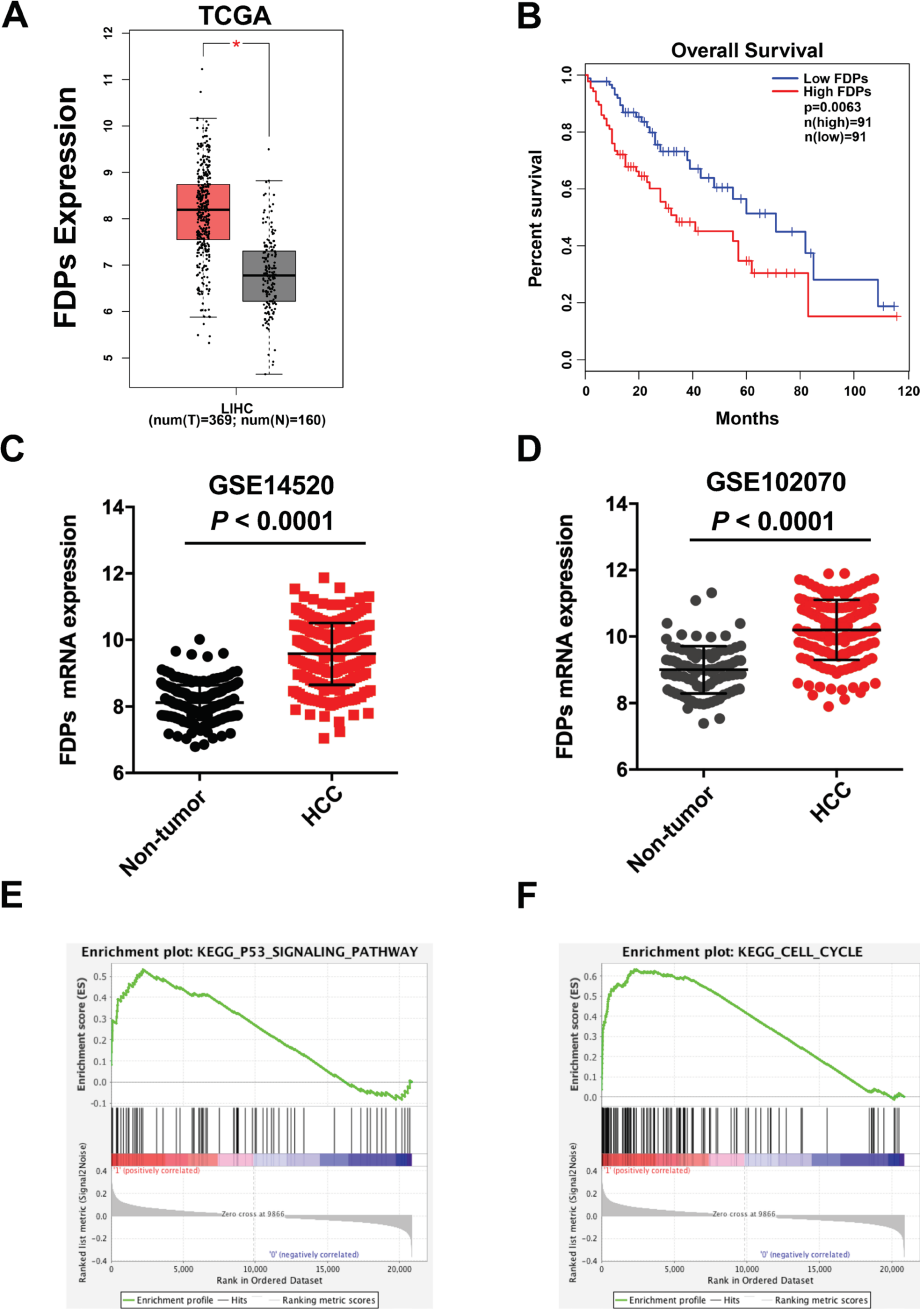
Bioinformatics analysis of FDPs expression in HCC. (A) FDPs expression in TCGA database. HCC tissue = 369, Normal tissue = 160. (B) Log‐rank (Mantel Cox) survival test of HCC patients based on the levels of FDPs expression (Low expression *n* = 91, high expression *n* = 81, *p* = 0.0063). (C, D) FDPs expression analyzed in adjacent non‐tumor and tumor tissue using GEO datasets (GSE14520 and GSE102070). (E, F) GSEA analysis showed FDPs expression is positively related to P53 signaling pathway and cell cycle pathway.

To investigate the clinical significance of FDPs in HCC patients, we analyzed the public TCGA database to find out the expression of FDPs is higher in HCC tissue comparing to normal control (Figure 1A). Furthermore, the overall survival analysis also indicated that those with high expression of FDPs inversely relate to low survival rate (Figure 1B). In this context, after analyzing the transcriptome data assembled from GEO database (GSE14520, GSE102070), the results indicated that the RNA expression of FDPs is higher in HCC (Figure 1C,D). These data showed that FDPs might play an oncogenic role in HCC. It is well known that cell proliferation plays an important role in the initiation of tumor formation. Therefore, we wonder what is the role of FDPs within the process of cell proliferation. From the GSE14520 and GSE102070 database, we conducted GSEA analysis to find out FDPs expression is positive related to the p53 signaling pathway and cell cycle process (Figure 1E,F), which indicating that FDPs also regulate the HCC cell proliferation in a p53 dependent manner. The results above suggested that FDPs may play an important role in the HCC progression by regulating HCC cell proliferation.

### 3.2 FDPs Induces HCC Cell Proliferation

**FIGURE 2:**
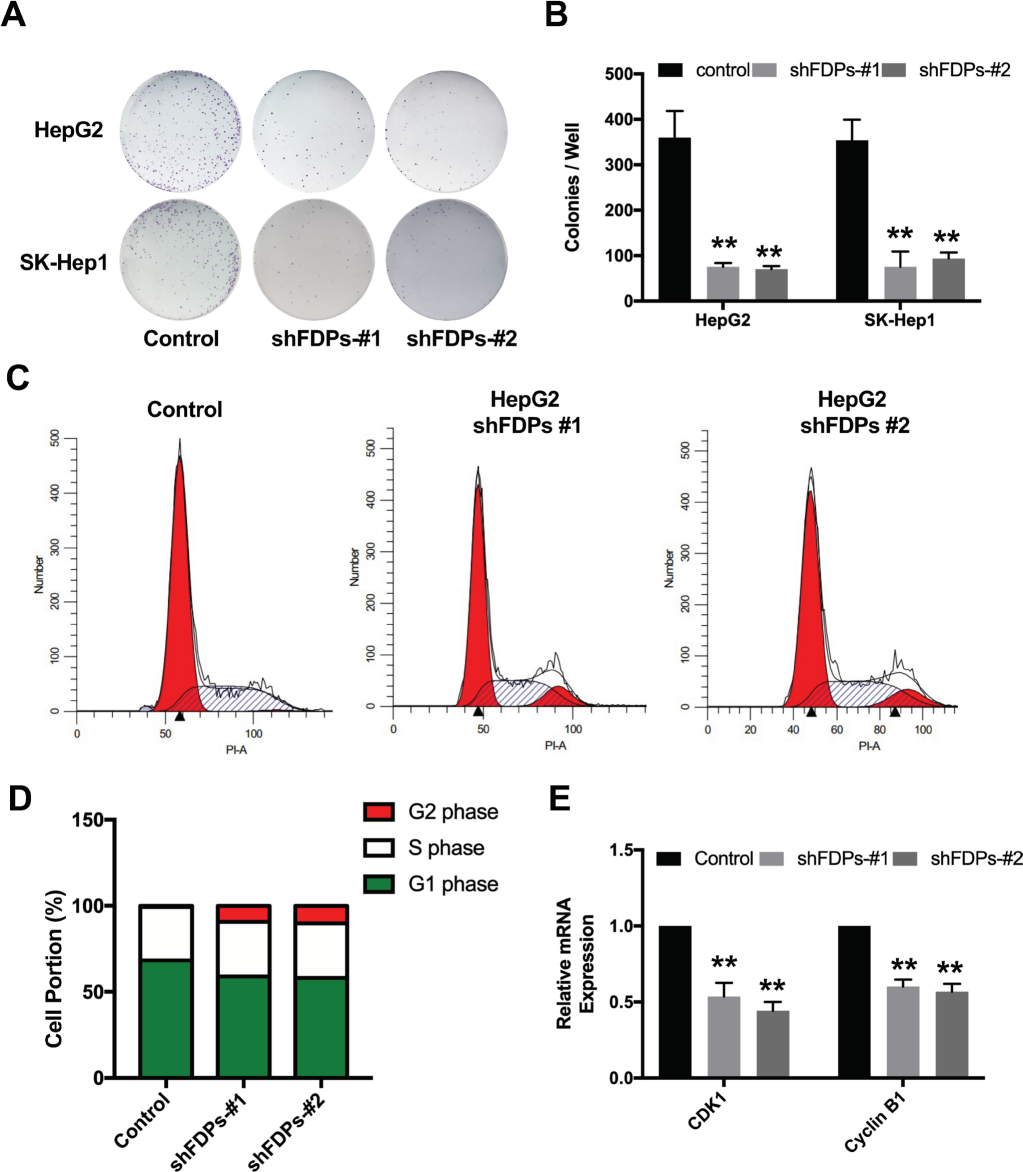
Effect of FDPs on cell proliferation in HCC cells. (A, B) Colony formation assay was performed with indicated FDPs knock‐down SK‐HEP1 and HepG2 cells. Representative images were displayed. (C) Cell cycle detection was performed using FDPs knock‐down HepG2 cells. (D) Statistical analysis of cell cycle results. (E) The RNA expression of cell cycle related genes. The statistical differences were calculated, *n* = 3. *p < 0.05 and **p < 0.01 vs. Control.

To further investigate the function of FDPs in regulating HCC proliferation, we conducted stable FDPs knockdown HCC cell line SK‐Hep1 and HepG2 using lenti‐virus. The colony formation assay indicated that the FDPs knockdown significantly reduced the cell colonies number and formation ability (Figure 2A,B). Since cell proliferation is closely related to cell cycle regulation, we further detected the cell cycle in FDPs knock‐down HepG2 cell. The results from cell cycle assay showed that FDPs knock‐down induced cell cycle arrest at G2 phase (Figure 2C,D). In this context, further RNA expression of CDK1 and Cyclin B1 within G2 phase was also down‐regulated after FDPs knock‐down (Figure 2E). These results suggest that FDPs play an important role in regulating cell cycle arrest in HCC cell proliferation.

### 3.3 FDPs Induces HCC Cell Apoptosis

**FIGURE 3:**
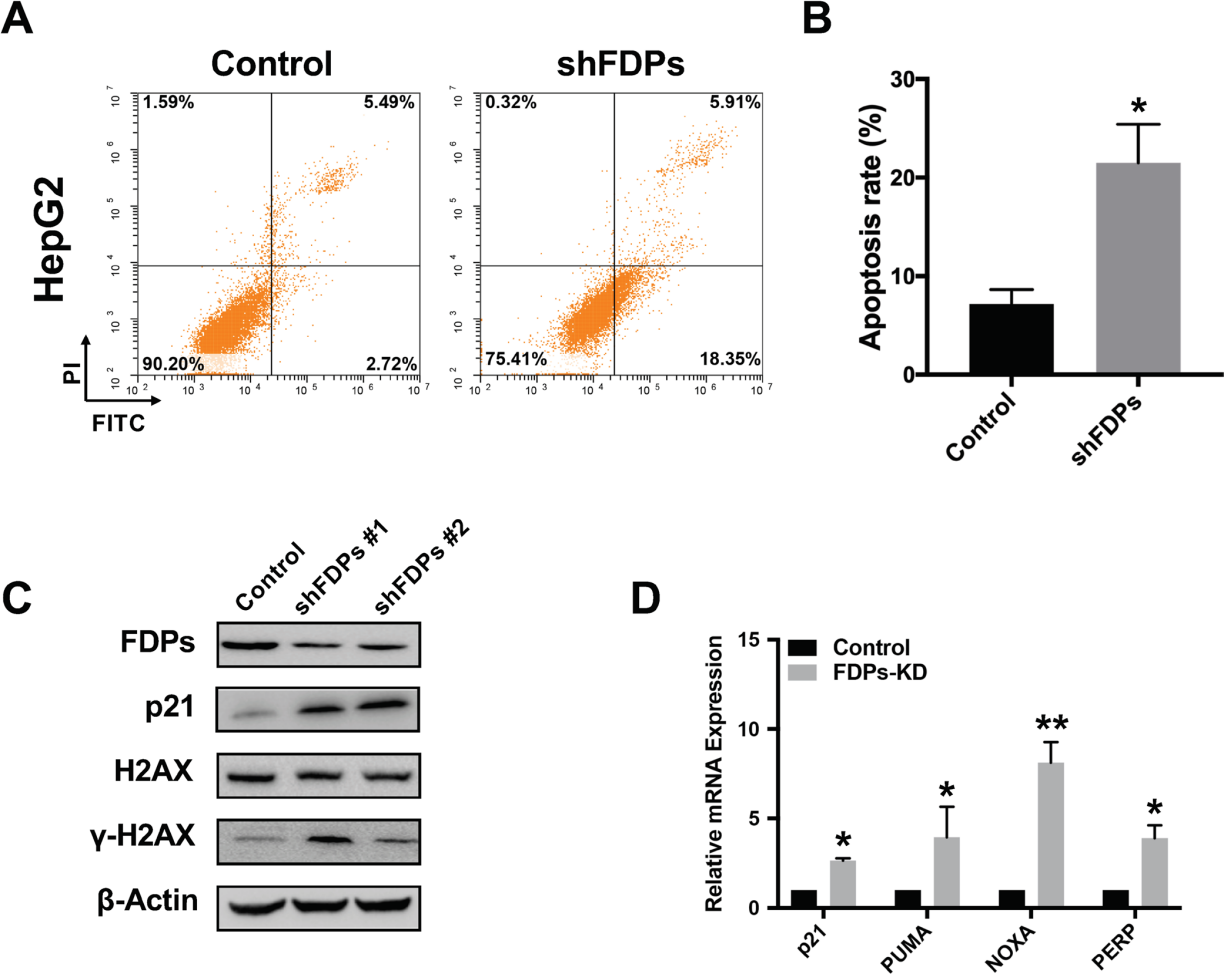
Effect of FDPs on cell apoptosis in HCC cells. (A) Cell apoptosis assay was performed and data was analyzed by FACS. (B) Quantification analysis of cell apoptosis data. (C) The change of DNA DSB related proteins was detected by WB. Representative images were shown. (D) The RNA expression of P53 targets (P21, PUMA, NOXA, PERP) was detected by RT‐qPCR. The statistical differences were calculated, *n* = 3. *p < 0.05 and **p < 0.01 vs. Control.

Other than cell proliferation ability, the down‐regulation of cell apoptosis is associated with HCC progression. Therefore, we conducted a cell apoptosis assay in FDPs knock‐down HepG2 cells. The results showed that the down‐regulation of FDPs expression induced cell apoptosis (Figure 3A,B). It is common knowledge that cellular apoptosis could be induced by DNA double strand break (DSB). In this context, we further found that FDPs knock‐down induced the expression of H2AX at Ser139 (γH2AX), which is the marker for DNA DSB in the genome (Figure 3C). Furthermore, the expression of P21 was also induced after FDPs knock‐down, which also confirmed that the cellular apoptosis was induced by DNA DSB (Figure 3C). In consistent with the results from GEO datasets analyzed above, down regulation of FDPs expression activated the expression of P53 target genes, such as P21, PUMA, NOXA and PERP (Figure 3D). These results further support the idea that FDPs serve as an oncogenic gene in HCC and targeting FDPs might be used as an alternative to induce HCC cell apoptosis.

### 3.4 Down Regulation of FDPs Inhibits HCC Growth In Vivo

**FIGURE 4:**
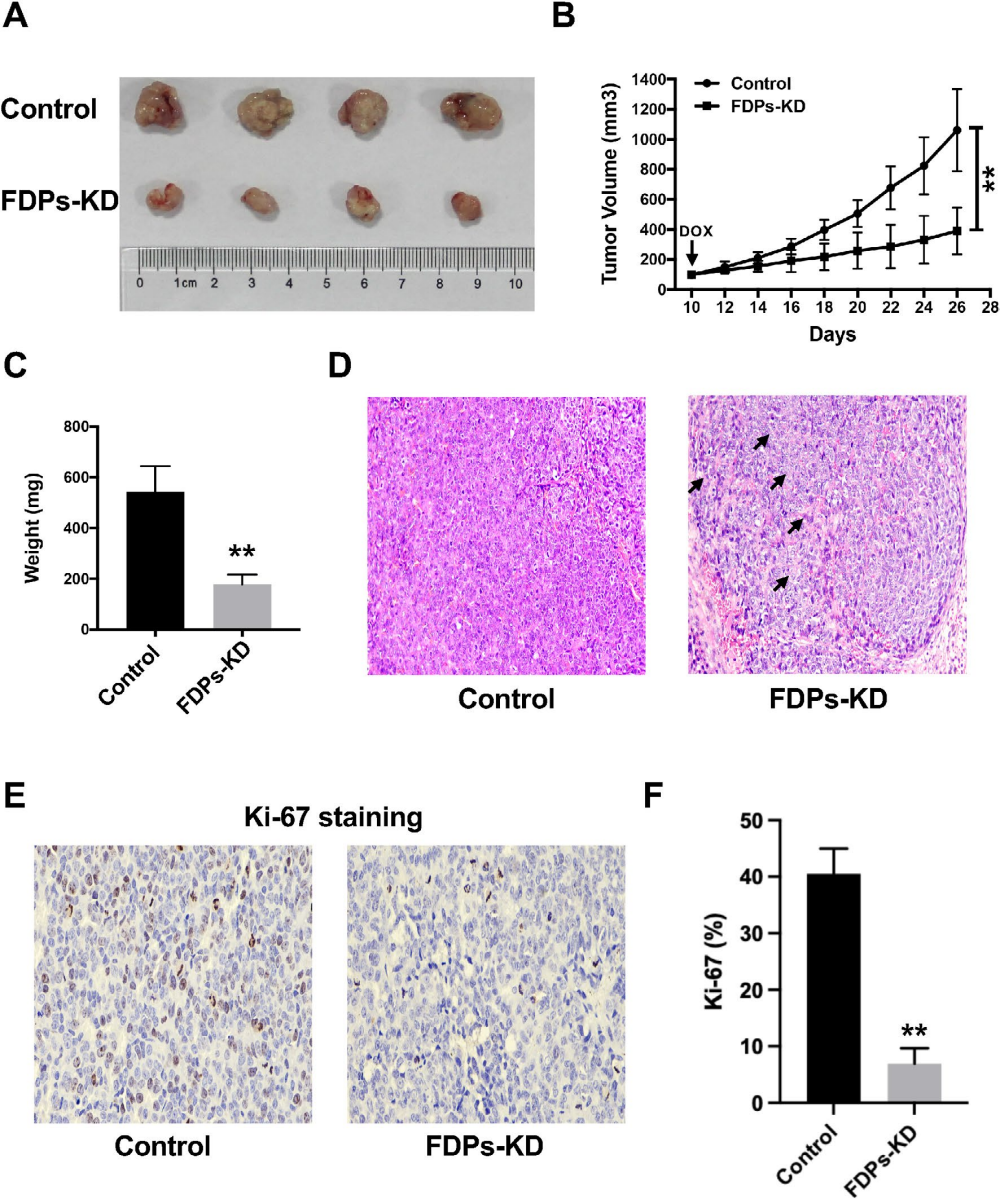
In vivo effect of FDPs in HCC. (A) Tumor xenografts model was established. HCC tumors were harvested and photographed. (B) The tumor growth curve was recorded at indicated time points in both groups. (C) Tumor weight was recorded in both groups. (D) H&E staining showed tumor necrosis in FDPs‐KD groups (black arrow), while tumor integrity was maintained in control groups. (E, F) Ki‐67 IHC staining in both groups was conducted. The Ki‐67 percentage was lower in FDPs‐KD group. The statistical differences were calculated, *n* = 4. *p < 0.05 and **p < 0.01 vs. Control.

In order to figure out the availabilities of targeting FDPs in vivo, we established a HepG2 subcutaneously tumor model. From the in vitro results, it is possible that knocking down FDPs may strongly suppress the growth of HCC tumor, which could not exhibit real tumorigenesis. Therefore, we generated an inducible stable knock‐down HepG2 cell line to conduct the in vivo analysis. After DOX was injected, the FDPs expression was induced to be down‐regulated (Figure S1A). Our data showed that low expression of FDPs had a suppressive manner in HCC tumor growth in vivo, as shown in Figure 4A,B. Furthermore, the tumor weight was significantly lower in the FDPs inducible knock‐down group (Figure 4C). To assess the pathological differences within the control and FDPs knock‐down groups, we further analyzed the H&E staining to show that aggressive tumor necrosis and less tumor integrity could be observed in the FDPs knock‐down group compared to control groups (Figure 4D). In addition, Ki‐67 staining to show the proliferation rate was also lower in FDPs groups from the immunohistochemical analysis results (Figure 4E,F). In consistent with the in vitro results, the in vivo analysis further supports that down‐regulating FDPs expression might be an effective way to slow the HCC tumor growth.

### 3.5 FDPs Knock‐Down Inhibits Multiple Oncogenic Pathways in HCC

**FIGURE 5:**
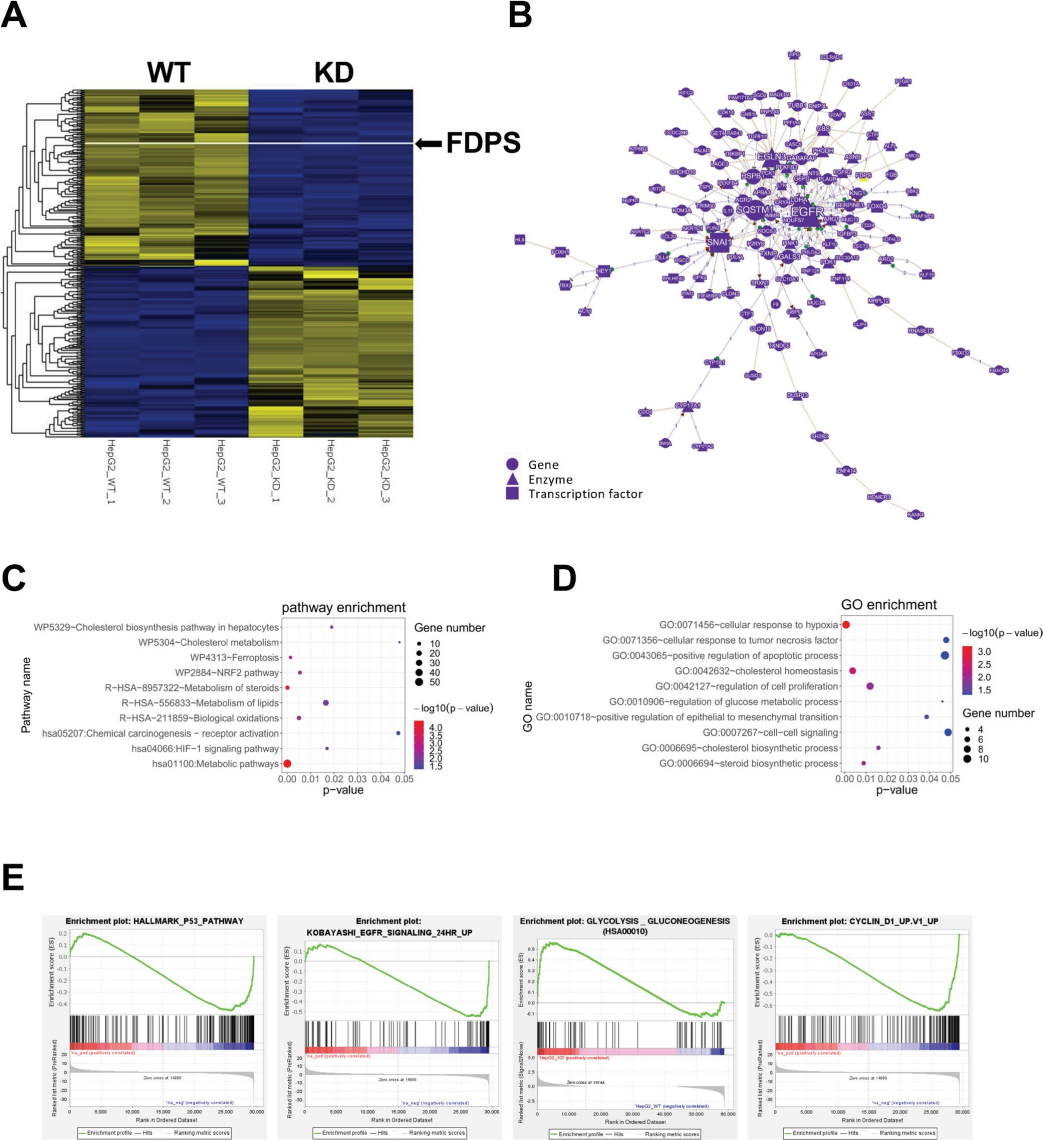
FDPs regulates multiple tumorigenic processes in HCC. (A) Heatmap of differentially expressed genes in FDPs‐KD HepG2. *n* = 3. (B) Regulatory networks of differentially expressed genes in FDPs‐KD HepG2. (C) Bubble diagram of partial significantly enriched GO biological processes for differentially expressed genes in FDPs‐KD HepG2. (D) Bubble diagram of partial significantly enriched pathways for differentially expressed genes in FDPs‐KD HepG2. (E) GSEA analysis of FDPs‐KD HepG2.

To reveal the biological effects and underlying mechanisms involved in the tumorigenic roles of FDPs in HCC, further RNA‐sequencing was performed within control and FDPs knock‐down HepG2 cells. 8527 genes were differentially expressed (adj *p* < 0.05) between control and FDPs knock‐down cells (Figure 5A), which FDPs were listed to confirm the accuracy of the sequencing results. Regulatory networks of differentially expressed genes in FDPs‐knockdown HepG2 were shown in Figure 5B. The size of the node represents the number of connections in this network. Genes are depicted as circles, enzymes as triangles, and transcription factors as squares; lines represent interactions, and the numbers on the lines represent the number of studies of interactions between genes. As we could tell, multiple oncogenic genes were involved, such as EGFR, SNAIL1, IGFBP3, FOXO4, G6PD, etc., representing metabolic pathways, epithelial–mesenchymal transition, and m6A modifications (Figure 5B). Furthermore, KEGG pathway enrichment analysis shows differentially expressed genes (DEG) were enriched in cancer pathways, such as the HIF‐1 signaling pathway, Ferroptosis pathway, and metabolic pathway (Figure 5C). Based on gene ontology (GO) enrichment analysis, we found that genes involved in cellular response to hypoxia and positive regulation of the apoptotic process were upregulated, while regulation of the glucose metabolic process and regulation of cell proliferation were downregulated (Figure 5D). In addition, Gene Set Enrichment Analysis (GSEA) further revealed that the pathways frequently up‐regulated during tumor promotion, P53 pathway, EGFR signaling pathway, glycolysis, and cell cycle processes were down‐regulated (Figure 5E). Above results further determined that multiple pathways and biological processes which promote cancer progression were down‐regulated when FDPs were knock‐down, which were associated with the retarded growth and cellular apoptosis observed in FDPs knock‐down HCC cells.

### 3.6 G6PD Related Glycolysis Might Be Regulated by FDPs

**FIGURE 6:**
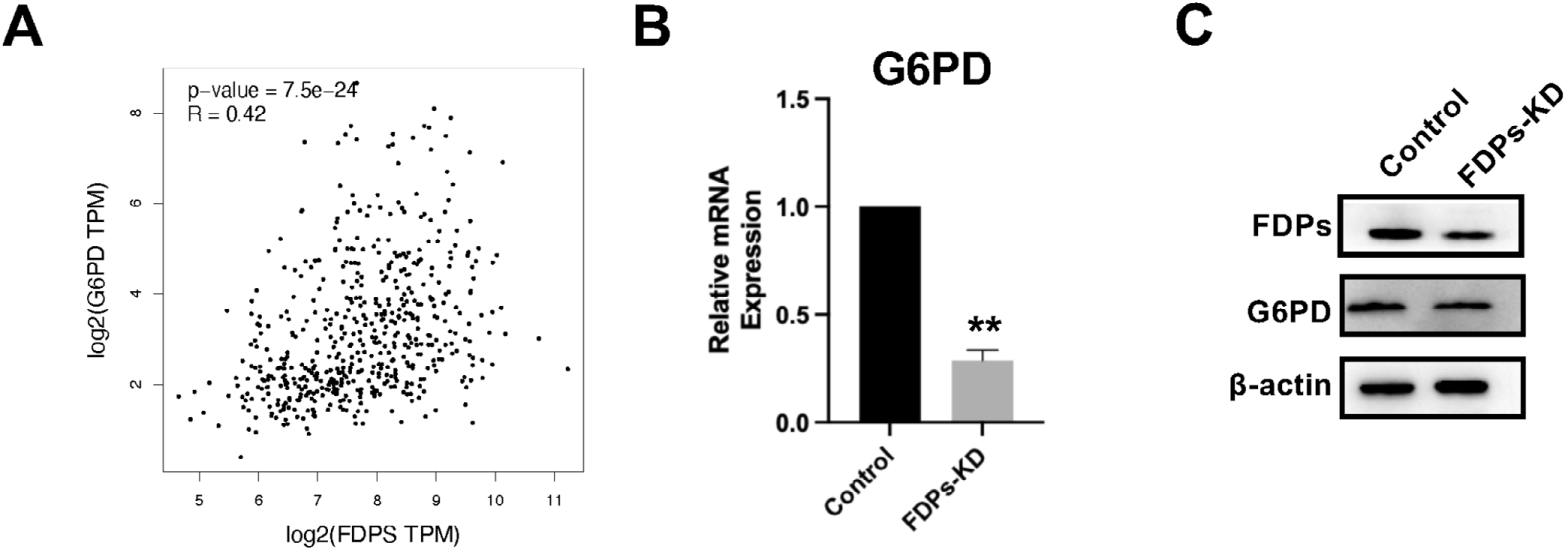
FDPs has a co‐relative connection with G6PD. (A) FDPs positively co‐relate to G6PD with *R* = 0.42. (B) RT‐qPCR verification of the G6PD expression in FDPs‐KD HepG2 cells. (C) WB detection of G6PD expression in FDPs‐KD HepG2 cells. The statistical differences were calculated, *n* = 3. *p < 0.05 and **p < 0.01 vs. Control.

Although multiple oncogenic pathways were involved in the process of FDPs downregulation, the glycolysis process was listed the most times, and the gene G6PD was closely interacted with FDPs, as shown from the sequencing results. Therefore, we further conducted the correlation analysis using GEO database to find out the expression of G6PD was closely related to FDPs expression (*R* = 0.42), as shown in Figure 6A. To verify what we found, RNA and protein expression of G6PD was detected in FDPs‐KD HepG2 cells, which supported our analysis (Figure 6B,C). In addition, we found protein–protein interaction between FDPs and G6PD in our immunoprecipitation assay (Figure S2). These results urge us to further investigate the relationship between G6PD and FDPs function in HCC.

### 3.7 G6PD Overexpression Reverses FDPs‐KD Biological Process

**FIGURE 7:**
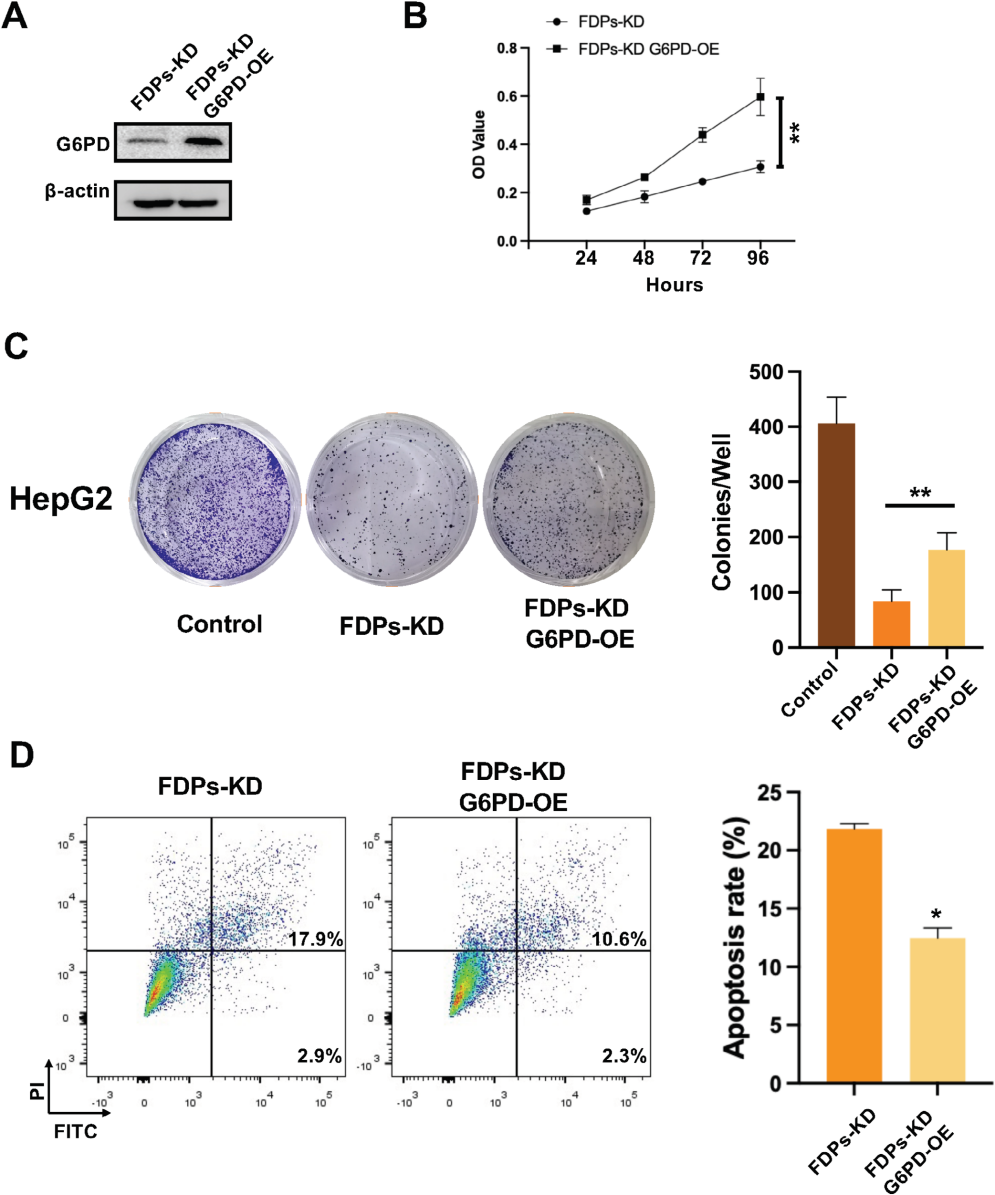
Overexpression of G6PD rescues FDPs‐KD induced cellular processes. (A) Confirmation of G6PD overexpression in FDPs‐KD HepG2 cells. (B) Proliferation of FDPs‐KD and FDPs‐KD G6PD‐OE cells was analyzed with CCK8 assay. (C) Colony formation of FDPs‐KD and FDPs‐KD G6PD‐OE cells was detected. Quantification analysis was performed. (D) Cell apoptosis of FDPs‐KD and FDPs‐KD G6PD‐OE cells was analyzed. Quantification analysis of apoptosis data was performed. The statistical differences were calculated, *n* = 3. *p < 0.05 and **p < 0.01 vs. FDPs‐KD group.

To further reveal the relationship between G6PD and FDPs, we conducted G6PD overexpression in FDPs‐KD HepG2 cells to determine whether FDPs‐KD related biological processes could be rescued. First, G6PD was overexpressed in FDPs‐KD HepG2 cells, which was confirmed by protein expression (Figure 7A). Following, a cell proliferation assay was performed; CCK‐8 analysis showed that G6PD overexpression, to some extent, reversed the cell proliferative activities (Figure 7B). Furthermore, a cell colony formation assay also supported the findings. After G6PD overexpression in FDPs‐KD cells, the cell colony formation ability was enhanced (Figure 7C). In addition, cell apoptosis was upregulated after G6PD overexpression in FDPs‐KD HepG2 cells (Figure 7D). Above supports the idea that G6PD was one of the downstream targets of the FDPs regulation network, which further proved that the glycolysis process might be closely related to FDPs function in HCC.

## 4 Discussion

HCC is one of the leading causes of tumor death around the world [1]. Although recent immunotherapies have had a breakthrough improvement in HCC treatments, chemotherapies are still the first‐class for HCC treatments [30]. However, prolonged treatment of chemotherapeutic drugs often leads to the emergence of drug resistance, whose underlying mechanism is currently unclear [31, 32, 33, 34]. Therefore, developing novel chemotherapeutic drug targets is urgent and also a necessity for HCC research and clinical treatment.

This study identifies a significant role of FDPs in HCC, underscoring their involvement in regulating cellular proliferation and apoptosis, which is essential for suppressing tumor cell growth and progression. By investigating FDPs expression and their involvement in key tumorigenic processes, this study has uncovered important features linking FDPs to metabolic homeostasis in HCC. While FDPs have been previously studied in cancers like bladder cancer and glioblastoma, this work provides a foundational understanding of their impact on HCC, offering a novel perspective on their potential as a therapeutic target. Through a series of experiments, we demonstrated that increased FDPs expression correlates with oncogenic properties, reinforcing their role in promoting tumor progression. In addition to confirming their pro‐tumorigenic role, our transcriptomic analysis uncovered a novel metabolic link, highlighting the pentose phosphate pathway enzyme (PPP) G6PD as a crucial downstream effector. The functional rescue through G6PD overexpression in FDPs‐deficient cells offers substantial mechanistic support for this axis, establishing FDPs as a pivotal regulator that integrates cholesterol biosynthesis with glycolytic activity. This revelation of the FDPs‐G6PD interaction provides a novel insight into metabolic cross‐talk in HCC, emphasizing FDPs as a promising therapeutic target.

Despite the valuable insights generated by this study, several limitations should be addressed to strengthen our understanding of FDPs and its role in HCC. The present data, encompassing RNA‐seq analysis and G6PD rescue experiments, robustly indicate this metabolic connection. However, we acknowledge that functional validation represents the essential evidence. To rigorously evaluate the metabolic consequences, Extracellular Acidification Rate (ECAR) and Oxygen Consumption Rate (OCR) assays will be conducted using a Seahorse XF Analyzer on control, FDPs‐KD, and FDPs‐KD/G6PD‐OE cells. Our hypothesis predicts that FDPs knockdown will reduce ECAR, signifying diminished glycolytic flux, and that this reduction will be restored through G6PD overexpression. Additionally, direct measurement of the NADPH/NADP+ ratio in these cell lines will be performed to verify that the FDPs‐G6PD axis influences the redox balance and the reductive biosynthetic capacity in HCC cells. Additionally, specific cholesterol metabolism assays, such as the enzymatic quantification of sterol intermediates, cholesterol uptake, export dynamics, and esterification will also provide a deeper understanding of how FDPs influence cholesterol metabolism and its downstream effects on lipid biosynthesis and energy production. Such analyses, in conjunction with cell‐based metabolic profiling, will provide a more detailed characterization of FDPs' role in metabolic reprogramming in HCC.

G6PD, as the rate‐limiting enzyme of the pentose phosphate pathway (PPP), contributes to tumor growth by supporting nucleotide biosynthesis and managing cellular redox balance [35, 36, 37]. Our rescue experiments and RNA‐seq data indicate that G6PD serves as a critical downstream effector of FDPs. Although the mevalonate pathway generates intermediates such as GGPP, which can influence small GTPase activity, our mechanistic investigation has uncovered a more direct pathway of regulation. Specifically, we have identified a protein interaction between FDPs and G6PD, as revealed through immunoprecipitation assays. This protein–protein interaction offers a compelling molecular mechanism underpinning the observed regulation. In future studies, we aim to elucidate the precise binding interface between these proteins and investigate the functional implications of this interaction on G6PD kinetics.

## 5 Perspective Remarks

In conclusion, this study identifies a novel oncogenic mechanism in HCC by demonstrating that FDPs act as a key regulator, directly interacting with G6PD to establish a functional link between the mevalonate and PPP. The FDPs‐G6PD axis constitutes an unrecognized metabolic regulation that highlights FDPs as a promising therapeutic target. The observed protein–protein interaction suggests that inhibiting FDPs could concurrently disrupt cholesterol biosynthesis, which is critical for tumor viability. By providing a mechanistic basis for targeting FDPs, this work establishes a compelling rationale for the development of specific FDPs inhibitors. These inhibitors could either serve as standalone agents or act synergistically alongside statin therapies to enhance antitumor efficacy. A combination strategy targeting both HMG‐CoA reductase and FDPs has the potential to deliver a coordinated effect on the metabolic machinery driving HCC proliferation and survival. Overall, our findings position FDPs as a promising target for therapeutic intervention in HCC, providing a basis for future studies to expand our understanding of its role in cancer metabolism.

## Author Contributions

Jingfeng Liu, Yisheng Zhu and Jiyang Lv: validation, investigation, data analysis, writing – original draft, writing – review and editing. Xiaohao Hu: investigation. Yan Zhong and Jingfeng Liu: conceptualization, validation, investigation, writing – original draft, writing – review and editing, funding acquisition, supervision.

## Funding

This work was supported byNational Natural Science Foundation of China, 82202987, 82203619, Shenzhen Medical Research Fund, A2403057and Shenzhen Science and Technology Innovation Program, JCYJ20220530154202005, JCYJ20220530160216037.

## Conflicts of Interest

The authors declare no conflicts of interest.

## Supporting information


**Figure S1:** Inducible KD of FDPs expression in HepG2 cells. (A) Indicated concentration of DOX was added to induce FDPs knock down. Quantification analysis was calculated.

**Figure S2:** Immunoprecipitation assay confirmed protein–protein interaction between FDPs and G6PD. (A) Immunoprecipitation assay was performed between FDPs and G6PD in HepG2 cells.

## Data Availability

The data that support the findings of this study are available from the corresponding author upon reasonable request.
