# Supplementary figures and images for: Farnesyl Diphosphate Synthase Promotes Proliferation of Hepatocellular Carcinoma Cells by Interacting With Glucose‐6‐Phosphate Dehydrogenase
Source: Cancer Med. 2026 Feb 10;15(2):e71620. doi: 10.1002/cam4.71620 (PMC12890576; doi:10.1002/cam4.71620)

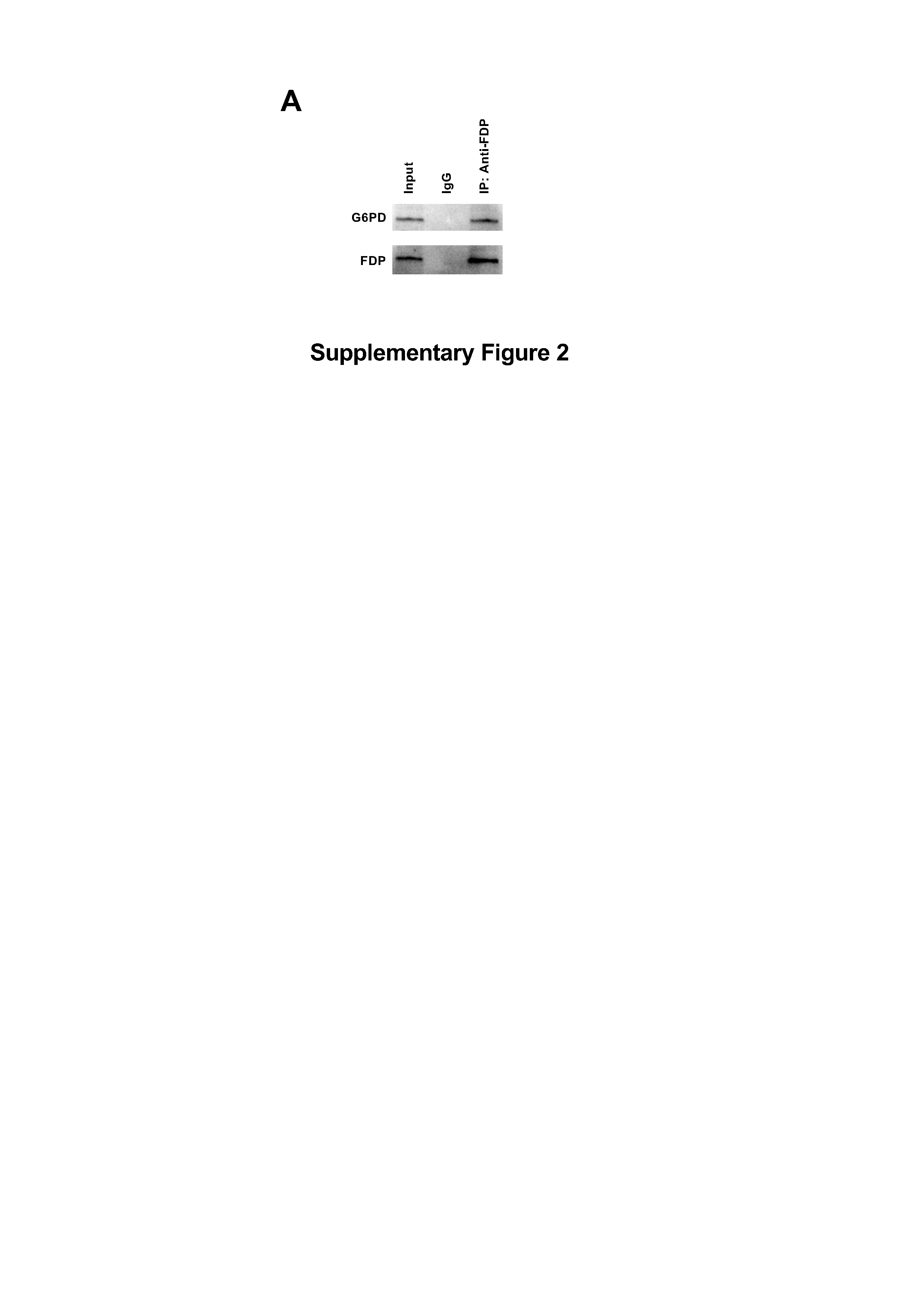

Supplement: Supplementary file 1 — Figure S1: Inducible KD of FDPs expression in HepG2 cells. (A) Indicated concentration of DOX was added to induce FDPs knock down. Quantification analysis was calculated. [file CAM4-15-e71620-s002.tiff]

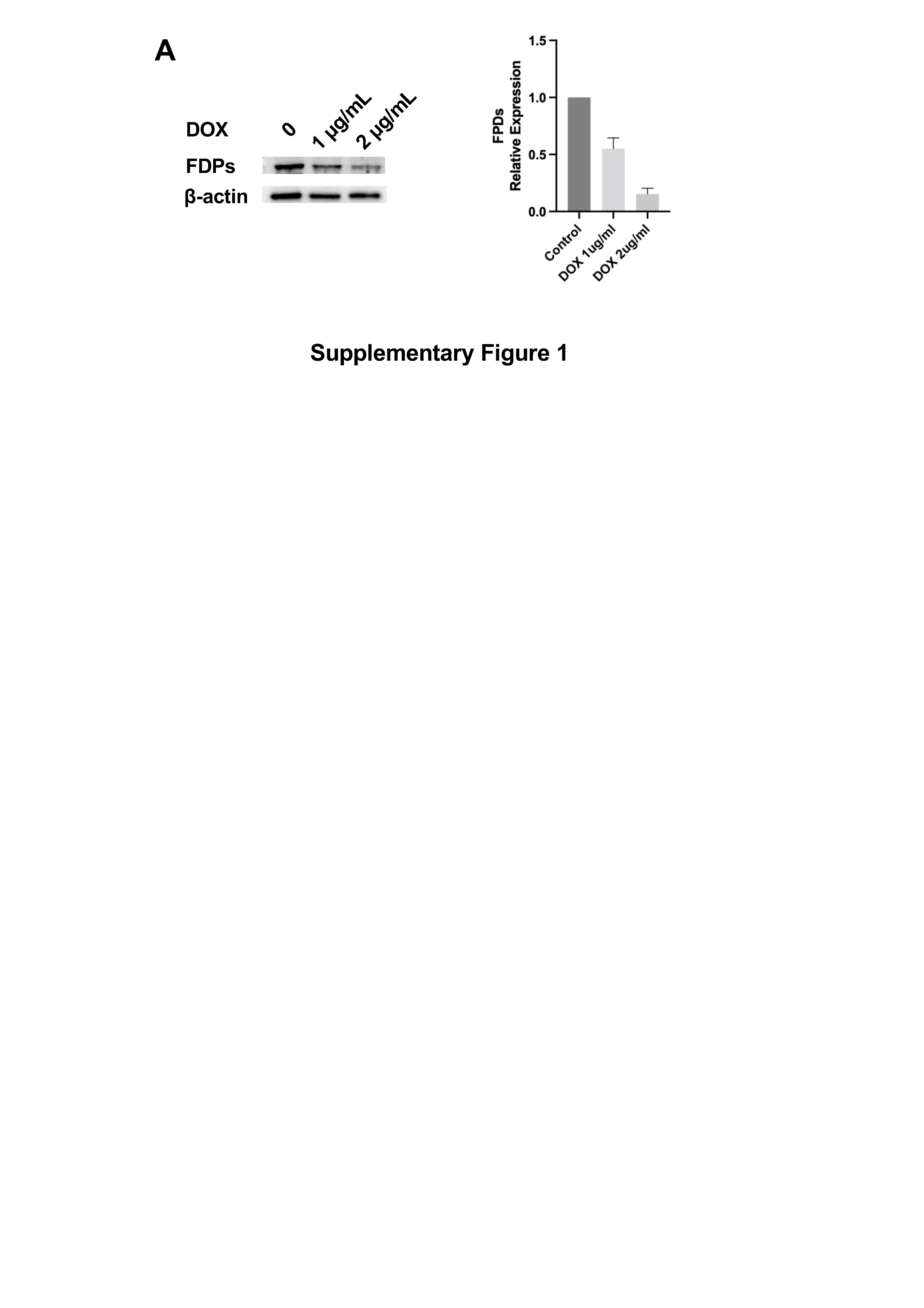

Supplement: Supplementary file 2 — Figure S2: Immunoprecipitation assay confirmed protein–protein interaction between FDPs and G6PD. (A) Immunoprecipitation assay was performed between FDPs and G6PD in HepG2 cells. [file CAM4-15-e71620-s001.tiff]
